# Dipole Determination by Polarimetric Spectroscopy Yielding the Orientation of Gold Nanorods

**DOI:** 10.1002/smsc.202400340

**Published:** 2025-01-16

**Authors:** P. Christian Simo, Annika Mildner, Dieter P. Kern, Monika Fleischer

**Affiliations:** ^1^ Eberhard Karls University Tübingen Institute for Applied Physics and Center LISA^+^ Auf der Morgenstelle 10 72076 Tübingen Germany

**Keywords:** analytical dipole model, gold nanorods, particle orientations, plasmonic nanostructures, polarizations, single‐particle spectroscopy

## Abstract

Plasmonic nanorods and other colloidal nanoparticles are widely used probes for enhanced spectromicroscopies. Precise knowledge of the angular orientation of individual nanostructures, respectively of their resonant modes, is required for purposes such as chiroptical spectroscopy or metasurfaces. However, noninvasive measurements of structures below the diffraction limit prove to be challenging. In this article, dark‐field spectroscopy requiring only four spectra in a simple microscope setup with a polarizer and an analyzer is employed in combination with an analytical dipole model to extract the orientation of the longitudinal dipolar mode of exemplary gold nanorods. This mode coincides with the azimuthal orientation of the colloid, and for irregularly shaped particles it can help to determine their dominant axis. The technique is demonstrated on multiple nanorods with varying aspect ratios. The spectroscopically determined orientations are compared to orientations extracted from electron microscopy images, resulting in standard deviations as low as ±2.4°. The method can be generalized to nanostructures with 3D orientations or multiple resonances.

## Graphical abstract

An all‐spectroscopic method is established to extract the azimuthal orientation of electric dipoles excited within gold nanorods of various aspect ratios. By using an analytical dipole model in combination with polarimetric measurements that analyze the interplay between the structure's eigenmodes, an excellent orientation precision of ±2.45° is obtained as confirmed by scanning electron microscopy.

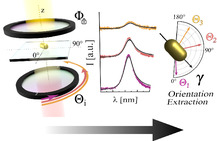

## 1 Introduction

The orientation information of a nanoparticle is of crucial interest, e.g., for biochemical and pharmaceutical research, which benefit from the alignment information in view of determining light polarization for chiroptical spectroscopy of optically active compounds.^[^ ^1^, ^2^, ^3^, ^4^ ^]^ Established metasurfaces exploit the orientation variation of *meta*‐atoms for wave front manipulation, thus directly affecting the polarization state of the transmitted light. Chiral metasurfaces, e.g., may be constructed from individual achiral structures.^[^ ^5^ ^]^ The structure tilt in each unit cell can be designed to control the phase shift using the Pancharatnam–Berry phase.^[^ ^6^, ^7^ ^]^ Applications within holography,^[^ ^8^, ^9^, ^10^ ^]^ photochemistry,^[^ ^11^, ^12^ ^]^ and sensing^[^ ^5^, ^13^, ^14^, ^15^, ^16^ ^]^ rely on high‐precision quality control to avoid negative effects through fabrication deviations.^[^ ^17^, ^18^ ^]^ Likewise, polarization‐encoded plasmonic color images that allow for storing multiple information in a single pixel through the shape and orientation of the nanoparticle constituents require precise orientation analysis.^[^ ^19^, ^20^ ^]^ In the emerging field of precisely stacked 2D van der Waals layer systems,^[^ ^21^ ^]^ one might follow the rotation angle in situ by reading out nanometric markers. Acquiring information about positioning and orientation of nanoparticles has been a common topic in optical, electron, and ion microscopy.^[^ ^22^, ^23^ ^]^ Despite being powerful and precise, electron and ion microscopy techniques may be prohibitive for further spectroscopic experiments due to the disadvantage of carbon deposition and the destructive nature of high‐energy ions, which may modify the nanostructures’ properties.^[^ ^24^ ^]^ Standard optical microscopy however is limited by spatial resolution.^[^ ^25^ ^]^ The in‐plane position of a single nanostructure can be determined from the center of its point spread function (PSF). In contrast, as the angular orientation of an examined nonspherical emitter is encoded in its emission, it is not directly apparent. The orientation of, e.g., dipolar emitters can be determined by examining their polarization angle‐dependent intensity pattern at the prominent mode frequencies.^[^ ^26^, ^27^ ^]^ This method is however time‐consuming and may limit the throughput of the analysis, since each particle needs to undergo several measurements with sufficiently small increments. Alternatively, a number of studies shows that the 3D orientation of single molecules can be extracted through wave‐optical modeling from defocused images, respectively, the shape of point‐spread functions,^[^ ^28^, ^29^ ^]^ back‐focal plane images,^[^ ^30^ ^]^ or imaging with higher‐order laser modes.^[^ ^31^, ^32^ ^]^ Recently, by using a polarization camera and laser illumination, researchers have determined the azimuthal orientation of molecules with a precision of 7.5° at low photon counts, increasing to 1° at high photon counts.^[^ ^33^ ^]^ Similar techniques were extended to gold nanoparticles.^[^ ^34^, ^35^, ^36^ ^]^ Already early on, it was demonstrated how the polarization dependence of the photothermal intensity of nanorods (NR) could be evaluated to extract the dipole orientations of their longitudinal and transversal surface plasmon resonance (LR, TR). The results yielded about 5% error compared to SEM images with a slight systematic offset.^[^ ^27^ ^]^ Colorimetric studies of the polarization‐dependent spectral modifications of NRs investigated by dark‐field spectroscopy allow for extracting both the LR and the TR mode of NRs,^[^ ^37^ ^]^ reaching precisions down to 2.3°,^[^ ^38^ ^]^ and were extended to further nanoparticle shapes.^[^ ^39^ ^]^ By hyperspectral dark‐field imaging, also the orientation of symmetric silver nanocubes could be extracted.^[^ ^40^ ^]^ Even nanomechanical spectroscopy of gold particles on membranes through polarization‐dependent absorption and resulting frequency tuning was demonstrated.^[^ ^41^ ^]^ Recent experiments in which dark‐field images of gold NRs (AuNRs) of various aspect ratios (ARs) were analyzed with a birefringent calcite optical component allowed for their in‐plane orientation determination with a precision of 4.6°.^[^ ^42^ ^]^ The use of predictive neural networks in image processing allows to determine the 3D orientation of asymmetric gold nanostars and AuNRs by referring to an image library and differential interference contrast microscopy with a ±20° standard deviation.^[^ ^43^ ^]^ Single‐shot high‐yield extraction of planar orientation information can also be achieved through dark‐field imaging of AuNRs with a Q‐plate and a polarizer. This allows the direct observation of a PSF modulation by the LR and TR modes with respect to the polarizing elements. Standard deviations of ±5° could be achieved for ARs >1.6. For lower AR (1.2–1.5), the deviation increases up to ±18° due to a more difficult reconstruction of the PSF.^[^ ^44^ ^]^ By manipulating the angular momentum of light, it was shown that 3D orientations of AuNRs could be imaged with high temporal resolution and an azimuthal precision of 2.5°.^[^ ^45^ ^]^

It is clear that emerging technologies and data science help to determine the orientation of nonspherical nanostructures nondestructively through optical imaging. They each have their advantages, uncertainties, and limitations. Here, we describe an alternative, easily available method to determine the azimuthal orientation of AuNRs of various ARs that requires only four dark‐field spectra in combination with an analytical extraction of the corresponding angles.^[^ ^46^ ^]^

## 2 Results and Discussion

**Figure 1:**
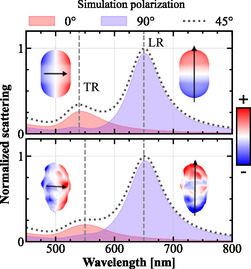
Simulation of (top) ideal and (bottom) nonuniform AuNR shape. The scattering spectra for all polarization angles were normalized by the maximum of the 45° polarization spectrum. The surface charges at $\lambda_{\text{TR}}$ and $\lambda_{\text{LR}}$ (vertical dashed lines) with the polarization along the short axis (0°) and long axis (90°) were used to determine the dipole moments $\vec{p}$. The dipole moments (black arrows) with their respective surface charges at $\lambda_{\text{TR}}$ and $\lambda_{\text{LR}}$ can be observed as insets on the left and right side of the spectra.

For the purpose of demonstrating the spectral orientation determination method, we first investigate the geometrical relation between the shape of a AuNR and the orientation of the dipole moments of its LR and TR. Conventionally these are aligned with the physical geometry of the AuNR such that the LR dipole is pointing along the long axis and the TR dipole along the short axis.^[^ ^27^, ^36^, ^44^, ^46^ ^]^ To further investigate how the dipole moment angle is influenced by the potential nonuniformity of a rod, an ideal AuNR and a similar AuNR with random nanoscale roughness were modeled and simulated in the MATLAB toolbox MNPBEM.^[^ ^47^, ^48^ ^]^ The simulation model was built to represent the analyzed sample and the optical setup as closely as possible, which includes a substrate and a particle coating of citrate stabilizers (see Experimental Section). Simulations were performed for polarization angles along the short axis (0°), long axis (90°), and at 45° to respectively excite the TR, LR, and both dipoles simultaneously. The calculated dipole moments at their maximum magnitude were plotted together with the corresponding surface charges of the ideal and nonuniform rods at the marked resonance wavelengths of the TR and the LR mode, $\lambda_{\text{TR}}$ and $\lambda_{\text{LR}}$ (see **Figure** **1**). As expected, the dipole moments follow the AuNR geometry in both versions with minute deviations. The largest deviation can be observed for the TR dipole moment that is tilted by 2°, which can be explained by the asymmetric surface roughness.

**Figure 2:**
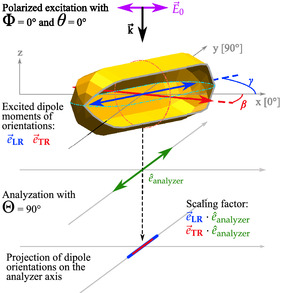
Illustration displaying the polarized illumination at normal incidence (θ = 0°) and polarization along the *x* axis (Φ = 0°) of a AuNR of arbitrary azimuthal orientation. The AuNR exhibits two distinct dipole moments (TR and LR) in the directions of ${\vec{e}}_{\text{TR}}$ (red) and ${\vec{e}}_{\text{LR}}$ (blue), which are defined by their azimuthal angle β and γ, respectively, as in Equation (1). The far‐field radiation of these dipoles passes an analyzing polarizing filter (${\hat{e}}_{\text{analyzer}}$), which (without loss of generality) has an azimuthal angle Θ = 90°. After the analyzer, the amplitude of the far‐field radiation is reduced by the dot product factor between the individual dipole moment and analyzer polarization. The dot‐dashed lines indicate the transversal (orange) and longitudinal (light blue) circumferences of the AuNR, where the AuNR exhibits rotational symmetry around the LR axis.

To extract the azimuthal orientation, one unpolarized and a minimum of three polarimetric measurements need to be conducted and fitted by an analytical dipole model established in previous work.^[^ ^46^ ^]^ There, it was demonstrated how the phase relation between the dipoles excited by linearly polarized light in an achiral nanostructure leads to local chirality in the near field. It could be shown that, through optical chirality flux,^[^ ^49^ ^]^ the chiral signature may also be detected in the far field. When the far‐field scattering is recorded under various analyzer angles, it is modified by the respective interplay between the two dipole modes. The scattering cross section could be reliably calculated compared to the corresponding measurements if the dipole moments and the polarizer angles are known.^[^ ^46^ ^]^ In the present work, the concept is reversed. Based on experimental spectra and the known polarizer angles, one can use the dipole model to fit the data and extract the dipole angles. This approach is demonstrated here with polarimetric measurements, i.e., analyzed signal, which shows the far‐field radiation of the TR and LR eigenmodes of the AuNR.^[^ ^50^ ^]^ The analysis is based on the fact that the spectral signature of the eigenmodes is shifting depending on the analyzer angle, which can be understood in the frame of the dipole model. The spectra are fitted to identify the phase relations between the eigenmodes. In general, the analytical dipole model shows the intensity of the electric far‐field radiation of dipoles excited with polarized light that is analyzed with a separate polarization orientation. The electric field of the excitation plane wave ‐ with a tilt angle θ relative to the surface normal and polarization angle Φ ‐ interacts with the free electron density of the nanostructure. The far‐field radiation of the excited dipoles is then analyzed with an additional polarizer at an angle Θ. To simplify the 3D model, the planar nanostructures are approximated as 2D objects with dipoles oriented in the *x–y* plane (see **Figure** **2**). With an analyzer that is positioned parallel to the *x–y* plane, the intensity can then be calculated as a function of the azimuthal polarizer, analyzer, and dipole angles (see Supporting Information). The orientations of the TR and LR dipoles are denoted with the respective azimuthal angles β and γ as shown in Equation (1).

(1) $$\begin{array}{ll} \text{TR}: & {\vec{e}}_{\text{TR}}=\cos\beta \cdot {\hat{e}}_{x}+\sin\beta \cdot {\hat{e}}_{y}\\ \text{LR}: & {\vec{e}}_{\text{LR}}=\cos\gamma \cdot {\hat{e}}_{x}+\sin\gamma \cdot {\hat{e}}_{y} \end{array}$$

(2) $$\begin{array}{ll} I_{p}(\lambda )= & A(d_{\text{TR}}^{2}(\lambda )\cos^{2}(\text{Φ}-\beta )\cos^{2}(\beta -\text{Θ})+\\ & d_{\text{LR}}^{2}(\lambda )\cos^{2}(\text{Φ}-\gamma )\cos^{2}(\gamma -\text{Θ})+\\ & d_{\text{TR}}(\lambda )\cos(\text{Φ}-\beta )\cos(\beta -\text{Θ})\cdot \\ & d_{\text{LR}}(\lambda )\cos(\text{Φ}-\gamma )\cos(\gamma -\text{Θ})\cdot \\ & 2\cos(\varphi_{\text{LR}}(\lambda )-\varphi_{\text{TR}}(\lambda ))) \end{array}$$

The intensity $I_{p}(\lambda )$ of the electric far‐field radiation, given in Equation (2), depends on the wavelength‐dependent relative mode intensities of the TR and LR dipoles $d_{\text{TR}}(\lambda )$ and $d_{\text{LR}}(\lambda )$ and phase shifts $\varphi_{\text{TR}}(\lambda )$ and $\varphi_{\text{LR}}(\lambda )$, respectively, as well as the azimuthal angles of the polarizer Φ, analyzer Θ, and those of the dipoles β and γ. The amplitude A is used as a scaling factor for the overall spectral shape. The line shapes of $d_{\text{TR}}(\lambda )$ and $d_{\text{LR}}(\lambda )$ are described by a skewed Lorentzian model, which is further detailed in the Experimental Section (Statistical Analysis) and Supporting Information.

The phase shift for a relative scattering magnitude $d_{i}(\lambda )$ of Lorentzian nature can be calculated by Equation (3), with $\Gamma_{\text{fwhm,i}}(\lambda )$ the corresponding full‐width at half‐maximum, where the index i denotes the TR or LR mode.

(3) $$\varphi_{i}(\lambda )=\arctan\left(\frac{\lambda \Gamma_{\text{fwhm,i}}(\lambda )}{\lambda_{\mathrm{res,i}}^{2}-\lambda^{2}}\right)$$

The first two terms of $I_{p}(\lambda )$ in Equation (2) display each dipole's magnitude in regard to its respective azimuthal angle relative to the polarizer and analyzer. They independently show the intensity variation of the two modes with respect to the polarizer and analyzer angles Φ and Θ. The projection of each dipole onto the analyzer axis is schematically illustrated in Figure 2. The third term shows the dipoles’ relative phase $\left(\Delta =\varphi_{\text{LR}}(\lambda )-\varphi_{\text{TR}}(\lambda )\right)$ to one another. The relative phase is crucial in describing the interaction between the two dipole modes, and together with the two polarizer angles it determines the overall spectral shape.

In case that one of the modes vanishes, either because the transversal mode decreases below a threshold where the spectral intensity eventually goes to zero ($d_{\text{TR}}^{2}(\lambda )\to 0$), or because the polarizer is oriented perpendicular to one of the dipoles (e.g., $\text{Φ}-\beta =\frac{\pi }{2}$), the analytical dipole model reverts to the scattering of a single dipole as shown in Equation (4).

(4) $$I_{p}(\lambda )=Ad_{\text{LR}}^{2}(\lambda )\cos^{2}(\text{Φ}-\gamma )\cos^{2}(\gamma -\text{Θ})$$

This will simply display the intensity modulation of the corresponding mode (e.g., LR) with varying analyzer angle Θ and a constant polarizer angle Φ. This way, the polar diagrams frequently used for determining the orientation of a single dipole may be recovered by choosing a set of analyzer angles and plotting and fitting the angle‐dependent intensities; however, the information on the second dipole would be lost.

In this study, Equation (2) is used to fit the recorded polarimetric data, with the amplitude *A* and LR angle γ as fitting parameters. The angular orientation of the AuNR in the *x–y* plane is directly related to γ, which the fitting procedure thus reveals. The TR angle β is set to be $\beta =\gamma -\frac{\pi }{2}$, assuming planar dipole orthogonality. Other known arguments such as the relative intensities $d_{\text{TR}}(\lambda )$ and $d_{\text{LR}}(\lambda )$ and phase shifts $\varphi_{\text{TR}}(\lambda )$ and $\varphi_{\text{LR}}(\lambda )$ were taken from the fit of the spectrum with unpolarized excitation. A fixed excitation polarization angle Φ=45° and three randomly selected analyzer angles Θ=[9°,106°,155°] were chosen for the polarimetric measurements, demonstrating that the method does not depend on any specific given angles. By evaluating different combinations of three angles, it was verified that any three values may be chosen. The error only increases if three closely spaced angles happen to be orientated close to the short axis, which can be avoided by selecting angles that span two quadrants. Generally, separate curve fittings for the three cases would yield slightly varying values for each parameter. In the present method however, by using a least‐squares fitting method, the polarimetric data of a single AuNR at separate analyzer angles Θ was fitted coherently to receive one single value for each parameter A and γ. For less than three polarimetric angles, the accuracy of the orientation determination was found to be insufficient, while for three angles a precision on the order of single degrees is achieved. The results can be further improved if spectra taken under more than three angles are included in the simultaneous fit, or several simultaneous fits over three different angles each are averaged.

**Figure 3:**
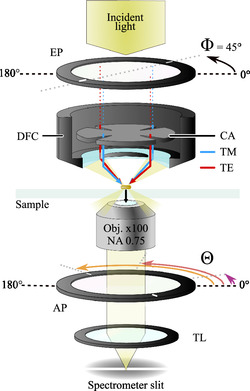
Optical setup schematic for polarimetric measurements. The unpolarized incident halogen light passes through the excitation polarizing filter (EP), which is locked at Φ=45°, leading into the dark‐field condenser (DFC) with NA 0.95–0.80. A cross aperture (CA) is introduced into the DFC and aligned to the EP. The alignment ensures that almost exclusively TM (blue arrow) and TE (red arrow) illumination is present at the focus of the DFC. The light scattered into the far field by the AuNR on the sample (black arrow) is collected by an oil‐immersion objective with NA 0.75. The collected far‐field radiation is analyzed by an additional polarizing filter (AP), which is rotated at a fixed angle of e.g., Θ=[9°,106°,155°]. The analyzed far‐field radiation is then finally focused by the tube lens (TL) to enter the spectrometer slit to record the spectrum. For unpolarized measurements, EP, CA and AP are removed from the optical path.

For the experimental extraction of the angular orientation of AuNRs, a colloidal solution of various ARs with two distinctly observable plasmonic modes was spin‐coated onto an indium tin oxide (ITO) coated glass substrate with a marker system (see Experimental Section). Unpolarized and polarimetric measurements were conducted for a total of 18 AuNRs without prior knowledge of their azimuthal position. For the polarimetric measurements, the conventional dark‐field spectroscopy setup that was used to obtain the unpolarized data was extended by an excitation polarizing filter at a fixed angle Φ and an analyzing polarizer filter at varying angles Θ to analyze the collected far‐field radiation. A cross aperture was added inside the dark‐field condenser to filter any incident light that cannot be converted to purely transverse magnetic (TM) and transverse electric (TE) polarization at the light focus (see **Figure** **3** and Experimental Section for more details). The AuNR positions were charted to ensure that the same particles could be investigated by correlative scanning electron microscopy (SEM). The unpolarized measurements were used to extract the wavelength‐dependent magnitudes $d_{i}(\lambda )$ and phase shifts $\varphi_{i}(\lambda )$ for each eigenmode of the AuNR, as these values are crucial for the determination of their orientation with the analytical dipole model. As introduced in Equation (1) and (2), the TR and LR eigenmodes can be described by the dipolar modes of two dipoles with relative scattering intensities $d_{i}(\lambda )$ and direction ${\vec{e}}_{i}$ each. Fitting the measurements under unpolarized excitation with two Lorentzian functions with wavelength‐dependent full‐widths at half‐maximum as introduced earlier (see equations in Statistical Analysis in the Experimental Section)^[^ ^51^ ^]^ yields the wavelength‐dependent curves $d_{\text{TR}}(\lambda )$ and $d_{\text{LR}}(\lambda )$ for the TR and LR modes with their respective amplitude A and resonance wavelength $\lambda_{\mathrm{res}}$. Their phase shifts $\varphi_{\text{TR}}(\lambda )$ and $\varphi_{\text{LR}}(\lambda )$ relative to the excitation wave can be extracted from the fits. It is well known that due to its higher refractive index, the presence of a substrate leads to a shift to longer wavelengths and a broadening of the NR resonances.^[^ ^52^ ^]^ While the spectra thus depend on the substrate properties, the in‐plane orientation of the dipoles remains unaffected. The dipole model makes no assumption about a substrate, but it describes any given two dipoles, which in the present case intrinsically contain the effects of the substrate. Therefore, the orientation analysis works independently of the given substrate.

**Figure 4:**
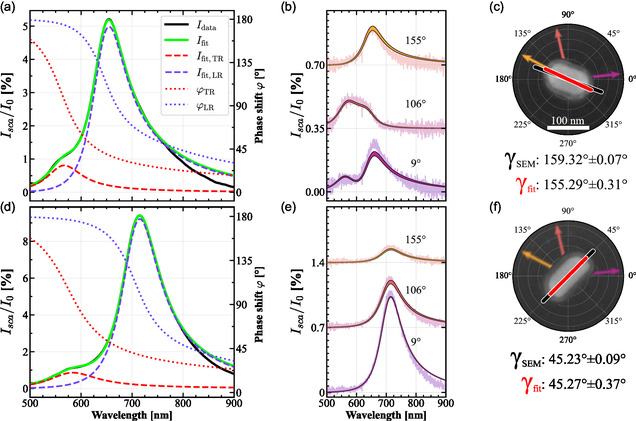
Measured a,d) unpolarized and b,e) polarimetric data with c,f) SEM micrographs of two AuNRs (particles *b* and *d* in Table 1). The data $I_{\text{data}}$ measured without polarizers with the double Lorentzian fits $I_{\text{fit}}$ show the two modes TR and LR with their respective fits ($I_{\text{fit,TR}}$, $I_{\text{fit,LR}}$) and phase shifts ($\varphi_{\text{TR}}$ and $\varphi_{\text{LR}}$). The stacked polarimetric data measured at analyzer angles 9°, 106°, and 155° also contain their fits, achieved with $I_{p}$ (Equation (2)) and modulated with the fitting errors $A_{\text{fit,err}}$ and $\gamma_{\text{fit,err}}$ from Table 1. The micrographs of the AuNRs contain the values and sketches of the SEM‐extracted angle $\gamma_{\text{SEM}}$ (black) and polarimetric angle $\gamma_{\text{fit}}$ (red), as well as the angles of the analyzing polarizer.

To prove the viability of this method, the obtained azimuthal orientation $\gamma_{\text{fit}}$ needs to be compared to the actual angle of the longitudinal axis. This geometric angle $\gamma_{\text{SEM}}$ can be extracted from SEM images. An image‐processing algorithm based on the OpenCV library was demonstrated on the simulated nonuniform AuNR and is used for SEM micrographs of the measured AuNRs to provide quantitative information about their orientation (see Supporting Information).^[^ ^53^ ^]^ In **Figure** **4**, the unpolarized and polarimetric data with individual fits can be observed for two exemplary particles (*b* and *d*, representing cases of relatively higher vs lower deviation between $\gamma_{\text{fit}}$ and $\gamma_{\text{SEM}}$, see **Table** **1**), each positioned in a separate rotation quadrant. Their corresponding polarimetric fitting curves were calculated with Equation (2) for the three analyzer angles Θ. Although there are some small deviations between the spectra and their fits due to the simultaneous fit of the three curves with a single set of parameters, the fitted angles are in good agreement with the corresponding SEM‐extracted angles as shown in the micrographs (Figure 4c,f). The fitting parameters and SEM‐extracted angles with their errors, as well as the aspect ratio and nonuniformity parameter χ (a measure for the deviations between the particles and their mirror image) are listed in Table 1. The definition of χ aswell as all other particle evaluations can be found in the Supporting Information.

**Table 1:** Fitting parameters and errors (standard deviations) for all 18 AuNR particles investigated with the polarimetric method compared to the SEM‐extracted angles and errors (see Supporting Information). Their individual aspect ratio AR and nonuniformity parameter *χ* are also listed. All errors were extracted from the respective covariance matrix of the fitting methods.

Particle	$A_{\text{fit}}$	$A_{\text{fit,err}}$	$\gamma_{\text{fit}}$	$\gamma_{\text{fit,err}}$	$\gamma_{\text{SEM}}$	$\gamma_{\text{SEM,err}}$	AR	*χ* [%]
a	0.392	±0.006	150.96°	±0.26°	150.05°	±0.06°	2.02	4.61
b	0.588	±0.006	155.29°	±0.31°	159.32°	±0.07°	1.87	3.10
c	0.428	±0.005	2.86°	±0.97°	2.56°	±0.21°	2.00	6.04
d	0.432	±0.003	45.27°	±0.37°	45.23°	±0.09°	2.28	2.48
e	0.452	±0.003	44.84°	±0.39°	46.89°	±0.04°	1.70	3.97
f	0.400	±0.003	36.85°	±0.46°	35.57°	±0.07°	1.37	6.43
g	0.479	±0.003	43.17°	±0.45°	47.82°	±0.05°	1.37	4.10
h	0.536	±0.008	110.91°	±0.49°	110.08°	±0.08°	2.30	4.14
i	0.480	±0.003	68.99°	±0.51°	73.56°	±0.09°	1.66	2.22
j	0.445	±0.003	71.40°	±0.55°	73.80°	±0.10°	2.11	2.31
k	0.450	±0.003	45.81°	±0.37°	48.93°	±0.09°	1.69	1.40
l	0.538	±0.003	136.96°	±0.28°	135.18°	±0.07°	1.73	4.80
m	0.463	±0.003	66.77°	±0.46°	66.40°	±0.05°	1.61	3.41
n	0.489	±0.003	74.75°	±0.65°	73.95°	±0.09°	1.78	2.55
o	0.460	±0.003	71.97°	±0.56°	75.67°	±0.07°	1.78	5.17
p	0.535	±0.007	105.31°	±0.51°	103.21°	±0.08°	1.92	2.28
q	0.507	±0.005	105.40°	±0.40°	101.79°	±0.09°	1.64	2.04
r	0.452	±0.003	25.85°	±0.84°	25.68°	±0.09°	2.09	2.73

Any remaining discrepancies can partly be explained by how the data was recorded and fitted. Generally, an offset of the polarization filters relative to the sample axes could play a role, as the precision for adjusting this angle is limited. Another possible cause may be TR modes that are not, as assumed, oriented by $\frac{\pi }{2}$ with respect to the LR, due to nanoscale asymmetries of the AuNRs. This was also observed in the presented simulations of a nonuniform AuNR. For this last reason, the nonuniformity parameter χ was extracted together with the aspect ratios. However, exemplary analyses showed that the rotational angle of the LR dipole remained unchanged when the TR dipole is freely fitted as well. As listed, the range of ARs and nonuniformities within the AuNR sample is quite broad (AR = 1.37–2.30, χ = 1.4–6.4%). Nonetheless, for the particles *b* and *d* one obtains angles $\gamma_{\text{fit}}$ of 155.29° and 45.27°, which deviate only by about 4° and 0° from their corresponding $\gamma_{\text{SEM}}$ of 159.32° and 45.23°. The usable range of ARs is only limited by whether the TR and LR mode can be clearly distinguished and separately fitted.

**Figure 5:**
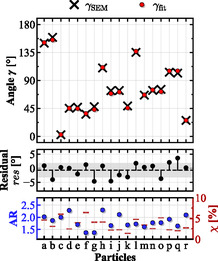
(Top) Scatter plot of the SEM‐extracted $\gamma_{\text{SEM}}$ (crosses) and polarimetrically extracted $\gamma_{\text{fit}}$ (dots) angles, taken from Table 1. (Middle) The residuals res between the two angles are plotted below with the average (dashed line) and the first standard deviation of ±2.45° (gray band). (Bottom) AR and nonuniformity parameter χ taken from Table 1.

Both $\gamma_{\text{fit}}$ and $\gamma_{\text{SEM}}$ for each particle (see Table 1) are summarized in **Figure** **5** together with the residual angle $\mathrm{res}=\gamma_{\text{fit}}-\gamma_{\text{SEM}}$. The $\gamma_{\text{fit}}$ angles coincide very well with the angles $\gamma_{\text{SEM}}$ and present a standard deviation of ±2.45°. The residuals range between 0.04° and 4.65° without an apparent correlation to the particles’ AR, as stated for other methods.^[^ ^43^, ^44^ ^]^ A principal component analysis (PCA) was conducted with a total of seven features (AR, χ, $A_{\text{fit}},\gamma_{\text{fit}},A_{\text{fit,err}},\gamma_{\text{fit,err}}$, mean squared error (MSE) of the unpolarized fit) to determine any statistical connection between each feature and the angle residuals res (see also Supporting Information). Vanishing correlations were observed for all parameters. The relatively highest weight could be attributed to the MSE of the unpolarized data residuals. The more exact the fit of the respective modes, the better the result of the polarimetric data can be fitted. Although the predictions of the in‐plane orientation yield good results already with a total of three spectra, by choosing more than three polarimetric spectra, the precision could be still further improved.

## 3 Conclusion

To conclude, we present an alternative method to determine the azimuthal orientation of AuNRs by spectroscopic means. With just four spectra per AuNR, the LR angle γ of a total of 18 arbitrarily oriented AuNRs could be determined using a comprehensive analytical dipole model, which reduces the standard deviation to a few degrees. In comparison, polarization scanning measurements often use more individual measurements to reach a comparable precision.^[^ ^54^, ^55^ ^]^ Considering the wide range of aspect ratios and low residuals between the polarimetrically determined $\gamma_{\text{fit}}$ and SEM‐extracted angles $\gamma_{\text{SEM}}$, the outlined method proved itself as a robust application with potential for automation. The analytical dipole model as presented in this letter is not limited to two dipolar modes and achiral structures and it can be extrapolated further to fit other particular nanogeometries. The approach may be of interest to any fields that rely on information about the orientation of metal nanoparticles, respectively, of the dipole moments within nanostructures, such as chiroptical spectroscopy for biomedical and pharmaceutical applications or the analysis of metasurfaces.

## 4 Experimental Section

4.1

4.1.1

##### Sample Preparation

Clean glass substrates were sputter‐coated with a 50 nm layer of conductive ITO, onto which gold markers were lithographically added to enable the charting of AuNRs. The hydrophobicity of the substrates was reduced by mild oxygen plasma treatment to increase surface wettability. An aqueous suspension of citrate‐stabilized AuNRs was spin‐coated directly from the stock solution onto the substrate leading to average particle–particle distances >4 μm between AuNRs. Further details on the fabrication process are available in the Supporting Information.

##### Measurements

Most SEM micrographs were taken at a Hitachi SU8030 SEM. Additional SEM micrographs were recorded at a Philips XL30 SEM.

All spectroscopic measurements were performed under dark‐field illumination at a modified inverted microscope (Eclipse Ti‐S, Nikon) (see Figure 3). A collimated 100 W halogen source was focused with a dry condenser (numerical aperture (NA): 0.80–0.95) onto the sample. The scattered signal was collected with a 100× oil‐immersion objective (CFI Plan Fluor, Nikon, NA adjusted to 0.75) and directed to the spectrometer (Shamrock SR‐303i, Andor) equipped with a CCD camera cooled to −60 °C (iDus 416 A‐LDC‐DD, Andor). The spectrometer's slit width was kept at 100 μm, ensuring a detection area of $1\times 1\;{\text{μm}}^{2}$ in combination with the mentioned objective. For unpolarized measurements, the setup remained unchanged. Polarimetric measurements were performed with additional optical and mechanical elements. Two ultra‐broadband polarization filters (WP25M‐UB, Thorlabs) and a 3D‐printed cross aperture were placed in the optical path (see Supporting Information). The excitation filter was placed before the condenser and fixed at 45° relative to the sample coordinate system, i.e., the stage axes. Inside the condenser, the cross aperture was placed on the condenser annulus. The cross aperture was aligned to the excitation filter angle such that the polarization axis is parallel to two opposing openings of the cross aperture, therefore allowing TM‐ and TE‐polarized excitation in the focal spot of the condenser. The analyzing polarizer was aligned with respect to the excitation polarizer.

As the spectroscopically determined angles will be compared to the SEM‐observed azimuthal angles, a systematic relative angle between the two methods has to be avoided. For this reason, a broad marker system is used to minimize the relative angles in both methods. During spectroscopy measurements, the markers were aligned to the x‐axis of the piezo scanning stage (*P*‐545.xR7, Physik Instrumente) in such a manner that the y position of a row of markers would not change if one would scan in x direction. For the SEM observations, the image was rotated with the scan rotation to align with the angle of the same row of markers.

All spectra were recorded by custom‐made software.^[^ ^56^ ^]^ Integration times of 20 s with three averages were kept for all recorded spectra. All spectra were background corrected and normalized to the excitation illumination. All unpolarized spectra were preprocessed with a Savitsky–Golay filter.^[^ ^57^ ^]^ Prior to initial measurements, a total number of 18 AuNRs were selected and charted on the sample with the help of the binary marker system under polarized dark‐field illumination. The selection criterion was solely that two modes (TR and LR) were distinguishable, which vary in intensity with the polarizer angle. Charting the AuNRs with a piezo scanning stage allowed for μm‐precision localization, which allows to identify the same individual AuNR for spectroscopic experiments and SEM imaging. All polarimetric data was fitted, and the errors were extracted from the covariant matrix by the least‐squares fitting method.

##### Simulations

Both an ideal AuNR and a AuNR with random nanoscale roughness and a similar AR that preserved the LR wavelength were modeled and simulated in the MATLAB toolbox MNPBEM.^[^ ^47^, ^48^ ^]^ The simulation model followed the experimental sample geometry and took into account the NA of the setup's objective. The AuNRs were positioned on a glass substrate with refractive index *n* = 1.5 and a 30 nm ITO layer (refractive index determined in‐house by ellipsometry) in the *x–y* plane with their long axis aligned to the *y* axis. The simulations included a coating layer on the AuNRs to take the layer of citrate stabilizers with *n* = 1.58 into account. The thickness of 10 nm was chosen such that the simulation matched the measured spectra as closely as possible. The simulations were performed with a plane wave at normal incidence for polarization angles along the short axis at 0°, long axis at 90°, and at 45° to respectively excite the TR, LR, and both dipoles simultaneously. The simulated complex surface charges $\sigma (\vec{r},t)$ were evaluated at the AuNRs’ respective resonance wavelengths $\lambda_{\text{TR}}$ and $\lambda_{\text{LR}}$ with $\sigma (\vec{r},t)=ℜ[\sigma (\vec{r})\cdot \exp(-i\omega t)]$ for an array of phases ωt∈[0,2π] to extract the time‐dependent dipole moments $\vec{p}(t)=\oint \sigma (\vec{r}\prime ,t)\vec{r}\prime dS\prime$.^[^ ^58^ ^]^

##### Statistical Analysis

The unpolarized spectrum and the polarimetric spectra of each AuNR were fitted with a skewed double‐Lorentzian function based on the amplitude $\tilde{A}$, the full‐width at half‐maximum $\Gamma_{\text{fwhm}}$, and the resonance wavelength $\lambda_{\mathrm{res}}$ of the respective resonance mode as shown in Equation (5), where the index i denotes either the TR or the LR mode. The skewness is introduced with Equation (6), which makes $\Gamma_{\text{fwhm}}(\lambda )$ a function of the wavelength, with the half‐width at half‐maximum of the non‐skewed Lorentzian $\Gamma_{0}$ and degree of skewness *σ*.

(5) $$I_{\text{Lorentzian,i}}(\lambda )={\tilde{A}}_{i}\frac{\Gamma_{\text{fwhm,i}}(\lambda )/2}{(\Gamma_{\text{fwhm,i}}{\left(\lambda )/2\right)}^{2}+{\left(\lambda -\lambda_{\mathrm{res,i}}\right)}^{2}}\;\hat{=}d_{i}(\lambda )$$

(6) $$\Gamma_{\text{fwhm,i}}(\lambda )=\frac{2\Gamma_{\text{0,i}}}{1+\exp[\sigma_{i}(\lambda -\lambda_{\mathrm{res,i}})]}$$

The MSE was extracted with $\text{MSE}=\frac{1}{n}\sum_{i=1}^{n}{\left(I_{\text{data}}-I_{\text{fit}}\right)}^{2}$.

A coherent least‐square fitting algorithm was used to simultaneously fit three polarimetric spectra recorded at three analyzer angles Θ=[9°,106°,155°] and constant polarizer angle Φ=45° with the analytical dipole model (see Equation (2)). The extracted parameters of the amplitude $A_{\text{fit}}$ and orientation $\gamma_{\text{fit}}$ and their standard deviations displayed in Table 1 were obtained by examining the inverse Hessian provided by the least square algorithm, and the residual covariance, which is the covariance of the output fitting function and the data.^[^ ^57^ ^]^ By multiplying the inverse Hessian with the factor of the residual covariance, we obtain the covariance matrix, which signifies the level to which the parameters vary. Since we evaluated two parameters (amplitude $A_{\text{fit}}$ and orientation $\gamma_{\text{fit}}$), this matrix is ultimately of the dimension 2×2. Extracting the diagonal of the covariance matrix and taking the square root of each element yields the standard deviation for the fitting parameters.^[^ ^57^ ^]^

The same principle was used for the SEM micrograph‐extracted angle $\gamma_{\text{SEM}}$ and its standard deviation displayed in Table 1, although in this instance the fitting was done with a simple Gaussian function (for more details see Supporting Information). The AR and nonuniformity parameter χ of each AuNR were extracted from the SEM micrographs. The angle residuals $res=\gamma_{\text{fit}}-\gamma_{\text{SEM}}$ with their standard deviation were separately plotted in the second row of Figure 5, since they were smaller than the symbol sizes in the main plot provided above.

For the ensemble of 18 AuNRs, each AuNR was characterized by one unpolarized spectrum and three polarimetric spectra, which yielded the following quantities: MSE of the unpolarized fit, fitted amplitude $A_{\text{fit}}$ with standard deviation, fitted orientation $\gamma_{\text{fit}}$ with standard deviation, extracted micrograph orientation $\gamma_{\text{SEM}}$ with standard deviation, AR, and nonuniformity parameter χ. All of these quantities were used as parameters for a PCA to correlate the factors to the angle residuals $res=\gamma_{\text{fit}}-\gamma_{\text{SEM}}$.

## Conflict of Interest

The authors declare no conflict of interest.

## Author Contributions

**P. Christian Simo**: conceptualization: (supporting); data curation: (lead); formal analysis: (equal); investigation: (lead); methodology: (equal); software: (lead); validation: (equal); writing—original draft: (lead). **Annika Mildner**: conceptualization: (lead); data curation: (supporting); formal analysis: (equal); methodology: (equal); supervision: (supporting); validation: (equal); writing—review & editing: (equal). **Dieter P. Kern**: conceptualization: (lead); methodology: (supporting); validation: (equal); writing—review & editing: (supporting). **Monika Fleischer**: project administration: (lead); resources: (supporting); supervision: (lead); validation: (equal); writing—review & editing: (equal). **P. Christian Simo** and **Annika Mildner** contributed equally to this work.

## Supporting information

Supplementary Material

## Data Availability

The data that support the findings of this study are available from the corresponding author upon reasonable request.
