# Supplementary material for: Dipole Determination by Polarimetric Spectroscopy Yielding the Orientation of Gold Nanorods
Source: Small Sci. 2025 Jan 16;5(6):2400340. doi: 10.1002/smsc.202400340 (PMC12168617; doi:10.1002/smsc.202400340)
Supplement: Supplementary file 1 — Supplementary Material [file SMSC-5-2400340-s001.pdf]

Supporting Information for 'Dipole Determination by Polarimetric Spectroscopy Yielding the Orientation of Gold Nanorods'

P. Christian Simo°, Annika Mildner°, Dieter P. Kern, and Monika Fleischer

° These authors contributed equally to this work.

Eberhard Karls University Tübingen
Institute for Applied Physics and Center LISA+
Auf der Morgenstelle 10, 72076 Tübingen, Germany

P. Christian Simo
Email Address: christian.simo@uni-tuebingen.de

Monika Fleischer
Email Address: monika.fleischer@uni-tuebingen.de

1 Sample Preparation

A marker system was fabricated by UV lithography on a glass slide with a conductive indium tin oxide (ITO) layer, onto which the gold nanorods (AuNRs) were spin-coated. For this purpose, a glass slide (22×22×0.1 mm^3) was thoroughly cleaned with an aqueous 2:1 [20%]KOH:H_2O_2 solution in an ultrasonic bath for 10 min. After rinsing and another 10 min ultrasonic bath with fresh deionized water, a 50 nm ITO layer was sputtered with a magnetron sputter coater (Univex 300, Leybold). A 1.5 µm thick photoresist maP-1215 (micro resist technology GmbH) was spin-coated onto the substrate and exposed in a mask-less aligner (µMLA system, Heidelberg Instruments) with a grid system. After development with maD-331/s (micro resist technology GmbH), a 30 nm gold film was deposited by thermal evaporation (Balzers BA-510) on the patterned photoresist. The sample was placed in an acetone lift-off bath to release the gold binary marker system on the ITO/glass substrate. The sample was treated with an oxygen plasma at 100 W for 30 s (Plasmalab 80 Plus, Oxford Instruments) to activate the surface and therefore to facilitate the spin-coating of an aqueous solution. The sample was then spin-coated with 50 µL of AuNR suspension (AC12-50-600-CIT, concentration 0.05 mg/mL, Nanopartz Inc.) at 2000 RPM for 60 s. The AuNRs were typically dispersed with distances >4 µm from each other.

2 Double Lorentzian fit

The spectra of gold nanorods (AuNRs) under unpolarized excitation were fitted with two Lorentzian functions as given in eq. (S1) to yield dipoles with the wavelength-dependent relative scattering intensities $d_1(\lambda)$ and $d_2(\lambda)$ for the transverse (TR) and longitudinal resonance (LR) modes with their respective amplitude A and resonance wavelength λ_{res}. Their phase shifts $\varphi_{\text{TR}}(\lambda)$ and $\varphi_{\text{LR}}(\lambda)$ relative to the excitation wavelength can be calculated according to eq. (S2). Presumably due to particle imperfections, a degree of asymmetry could be observed in unpolarized measurements. An asymmetric line shape could be fitted by assigning a wavelength dependency to the full-width at half maximum (fwhm) parameter, which is used for both Lorentzian and phase shift equations.[1]

The wavelength-dependent fwhm $\Gamma_{\text{fwhm}}(\lambda)$ is given in eq. (S3), where $2\Gamma_0$ is the fwhm of the non-skewed Lorentzian and σ the factor of skewness.

$$I_{\text{Lorentzian}}(\lambda) = A\frac{\Gamma_{\text{fwhm}}/2}{(\Gamma_{\text{fwhm}}/2)^2 + (\lambda - \lambda_{\text{res}})^2} \quad \hat{=}\, d_{\text{i}}(\lambda) \tag{S1}$$

$$\varphi(\lambda) = \arctan\left(\frac{\lambda\Gamma_{\text{fwhm}}}{\lambda_{\text{res}}^2 - \lambda^2}\right) \tag{S2}$$

$$\Gamma_{\text{fwhm}}(\lambda) = \frac{2\Gamma_0}{1 + \exp\left[\sigma(\lambda - \lambda_{\text{res}})\right]} \tag{S3}$$

3 Polarized light after dry condenser and cross aperture

To calculate the excited far-field intensity as a function of the azimuthal polarizer, analyzer, and dipole angles with eq. (5), the full 3D dipole model from Ref.[2] was simplified by the following assumptions. The excitation was fixed to normal incidence, and only dipoles oriented in the x-y plane were probed. The analyzer angles were defined in a plane parallel to that of the dipoles.
In the experiment, light passing through the dark-field condenser is incident under an angle. However, an additional cross aperture at the ring annulus of the dark-field condenser, which is aligned to the polarizer, ensures that only TM-polarized and TE-polarized light with in-plane electric field components parallel to the polarizer direction can pass (see Figure 3).[2] The analyzing polarizer installed in a piezo rotation mount (ELL14K, Thorlabs) was placed directly after the objective. The rotation mount could be controlled by the accompanying software with a 0.01° precision. By opening the aperture of the objective beyond the numerical aperture of the condenser, the full transmitted source signal could be gathered to adjust the cross aperture. By varying the angle of the analyzing polarizer, the recorded source intensity was minimized for the cross-polarization condition of both polarizers, which were thus aligned to each other.
In the focal spot of the condenser, the TE-polarized electric fields correspond to the ones obtained under perpendicular illumination. For TM-polarization, out-of-plane excitation is neglected since the in-plane fields are dominant, and the nanorods have a limited height. The assumption for the analyzer can be fulfilled by placing the analyzing filter orthogonally in the optical path of the objective that is used to collimate the far-field radiation of the dipoles.

4 Extracting the AuNR information from SEM images

This section describes the extraction of a AuNR's angle, aspect ratio (AR), and non-uniformity from scanning electron microscopy (SEM) images.
Consider a non-uniform AuNR shape as shown in the main text (see Fig 1, bottom), which will serve as an artificial SEM micrograph. The outline of the shape can be extracted from the normalized grayscale image with OpenCV library.[3] By finding the contour and thus also the geometric center of the arbitrary AuNR, one can extract its shape S_{arb}. To start the algorithm, the extracted shape and its horizontally mirrored copy S_{flip} are overlapped at their geometric center (see Fig. S1 (a, b)). When the images are rotated by the angle $\pm\alpha_{\text{i}}$ by values ranging from 0 to $\frac{\pi}{2}$ in opposite directions around their geometric

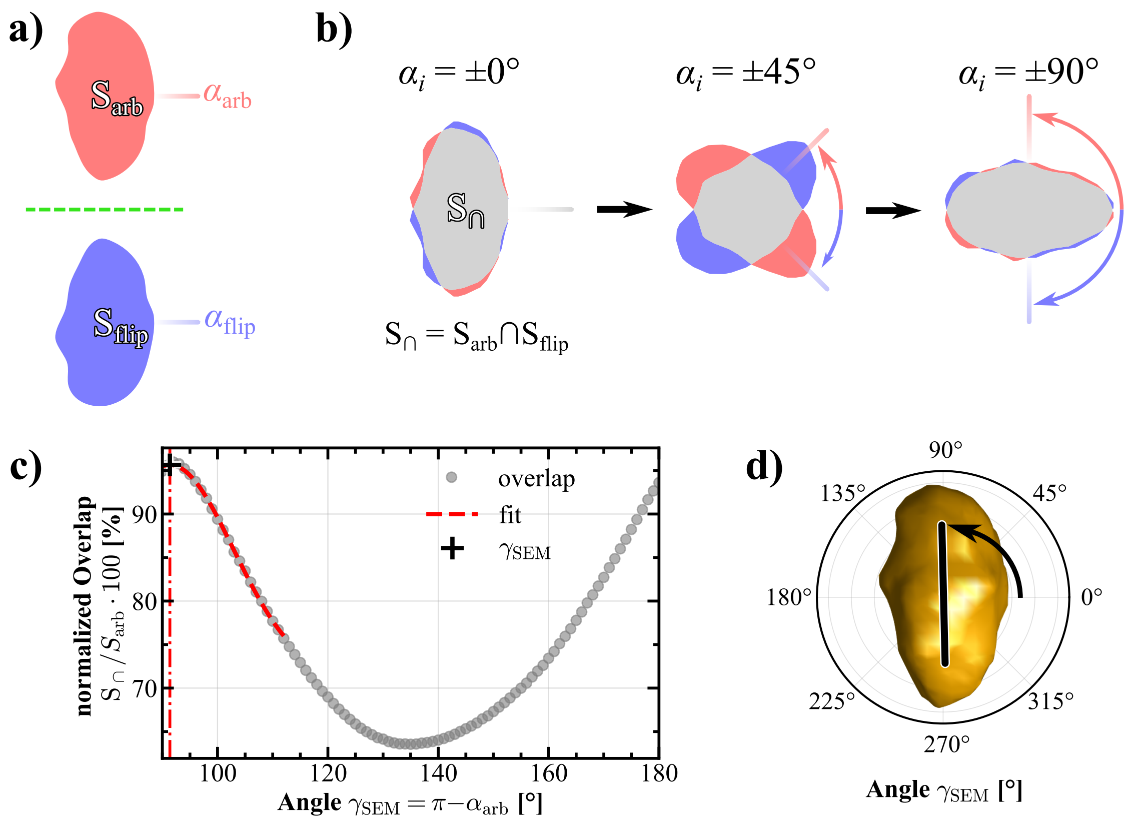

Figure S1: Visualized algorithm in which (a) an arbitrary shape S_{arb} and its mirrored version S_{flip} (b) are overlapped at their geometric center and rotated counterclockwise and clockwise, respectively, by the angle α_i. (c) During the rotation, the overlap $S_\cap$ is tracked for each angle, which then can be fitted to extract the longitudinal angle γ_{SEM} with the criteria stated in eq. (S4). This algorithm can be applied to determine (d) the orientation angle of a AuNR, the outlines of which were extracted from SEM micrographs.

center from their initial unknown orientations γ_{SEM}, the overlapping area of both shapes normalized by the particle area, $S_\cap / S_{arb}$, can be recorded as a function of the rotation angle. Let the rotation direction for S_{arb} be counterclockwise ($\alpha_{arb} = +\alpha_i$) and for S_{flip} be clockwise ($\alpha_{flip} = -\alpha_i$). For a AuNR, which has two symmetry axes, over the relative angle of $2\alpha_i = \pi$ a curve is generated that reveals one specific angle α of maximum overlap when the longitudinal axes of S_{arb} and S_{flip} are aligned, see Fig. S1 (c) (respectively two maxima at 0° and 90° if the AuNR is oriented along one of the coordinate axes, in which case the first maximum is chosen). This angle expresses the rotation from their initial orientations necessary to maximally overlap S_{arb} and S_{flip}. It represents the longitudinal angle as exemplarily measured and displayed in Fig S1 (d).

The unknown orientation γ_{SEM} can now be extracted by differentiating between two cases as stated in eq. (S4). Rotating S_{arb} counterclockwise by $+\alpha$, the long axis of $S_{arb}(\gamma_{SEM} + \alpha)$ will be positioned either horizontally or vertically, depending on the quadrant in which γ_{SEM} is located. For the horizontal case (along 180°), the longitudinal angle γ_{SEM} is the difference between 180° and the angle necessary for maximum overlap α ($\gamma_{SEM} = 180° - \alpha \equiv \pi - \alpha$). In the vertical case (along 90°), $\gamma_{SEM} = 90° - \alpha \equiv \frac{\pi}{2} - \alpha$. After evaluating the initial orientation γ_{SEM}, the particle and the orientation angle can be

visualized as seen in Fig. S1 (d).

$$\text{longitudinal angle } \gamma_{\text{SEM}} = \begin{cases} \pi - \alpha & \text{, for horizontal case} \\ \frac{\pi}{2} - \alpha & \text{, for vertical case} \end{cases} \tag{S4}$$

The aspect ratio (AR) is extracted from the AuNR axes in the situation of maximum overlap. A non-uniformity parameter χ (eq. (S5)) is introduced to quantify the deviation of a colloidal AuNR from its ideal shape, and to investigate whether the non-uniformity of a particle influences the correlation between the orientation of the longitudinal dipole moment and the axis determined from SEM images. As seen in Fig. S1 (b), the gray area represents the overlap between S_{arb} and S_{flip}, which is surrounded by areas with no overlap (red and blue). The areal percentage of the single (red *or* blue) non-overlapping area compared to the particle's area is evaluated at the angle of highest overlap, which illustrates the 2D non-uniformity of the particle.

$$\chi = \left(1 - \frac{S_{\cap}}{S_{\text{arb}}}\right) \cdot 100\% \tag{S5}$$

5 Principal component analysis

A principal component analysis (PCA) was conducted with a total of seven features (AR, χ, mean square error of the unpolarized fit, fitted parameters A and γ, and their errors) to determine the weight of each feature in regard to the angle residuals *res*. The PCA allows a statistical glimpse of the connections between the angle residuals and the aforementioned features. Vanishing correlations were observed for all parameters. A low weight could be found for the particles' AR towards the principal components. According to the PCA, the non-uniformity may have a larger impact on the distribution of the residuals. However, the relatively highest weight could be attributed to the mean square error (MSE) of the unpolarized data residuals. The MSE was determined with the residuals of the unpolarized data and its double asymmetric Lorentzian fitting curve, MSE $= \frac{1}{n}\sum_{i=1}^{n}(I_{\text{data}} - I_{\text{fit}})^2$. This influence is due to the dependency of the polarimetric fit on the extracted mode magnitudes and phase shifts. The lower the value of the MSE of the fit of the unpolarized spectrum, the better polarimetric data can be fitted.

6 Data overview for all investigated AuNRs

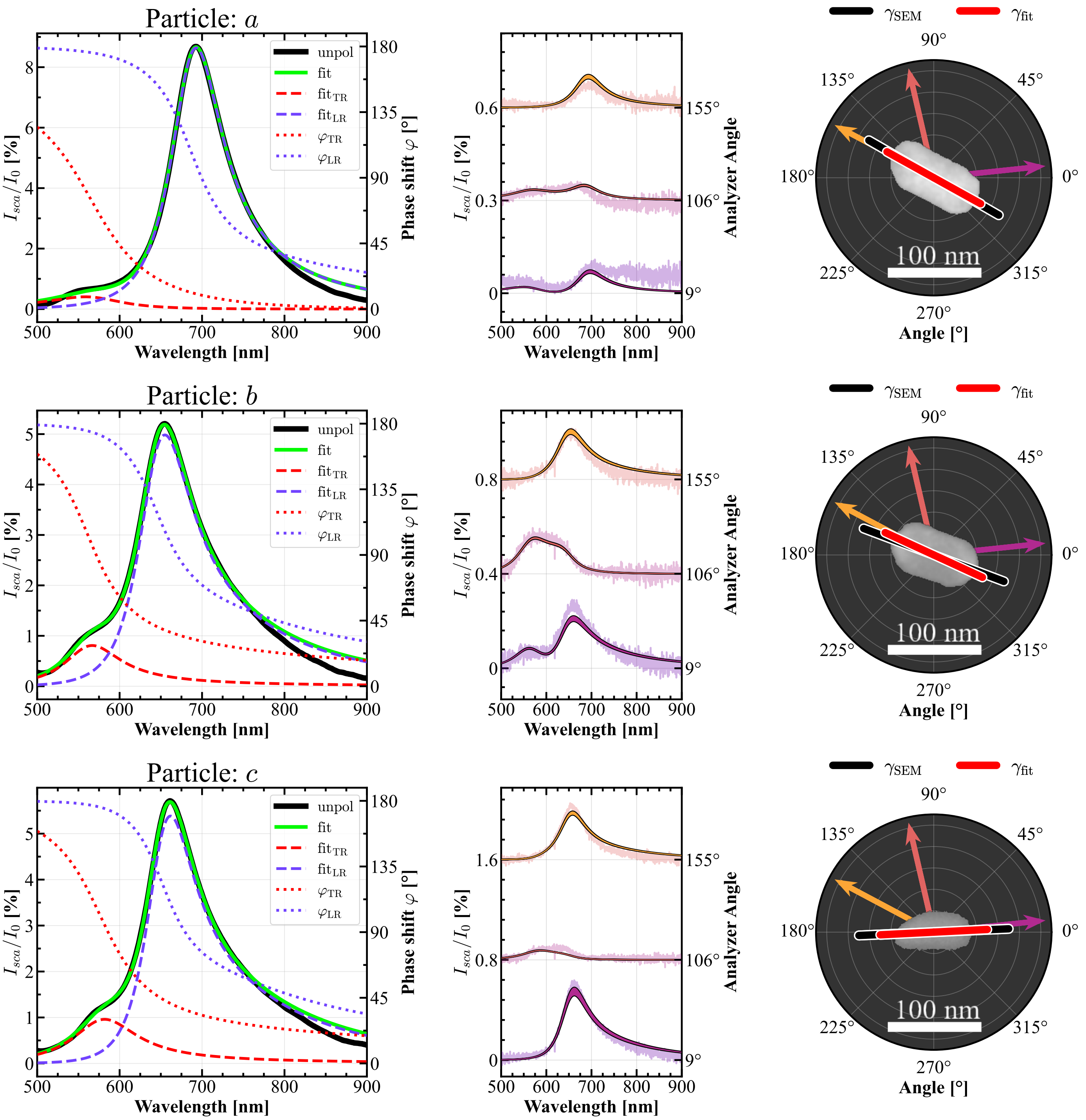

Figure S2: Particles $a-c$: (left) Unpolarized spectra with their respective double Lorentzian fit and dipole phase responses, (center) polarimetric measurements at the analyzer angles 9°, 106°, and 155°, and (right) the angles γ_{SEM} and γ_{fit} determined by SEM micrograph evaluation with the presented algorithm and cohesive fitting of the polarimetric measurements overlaid with the SEM particle.

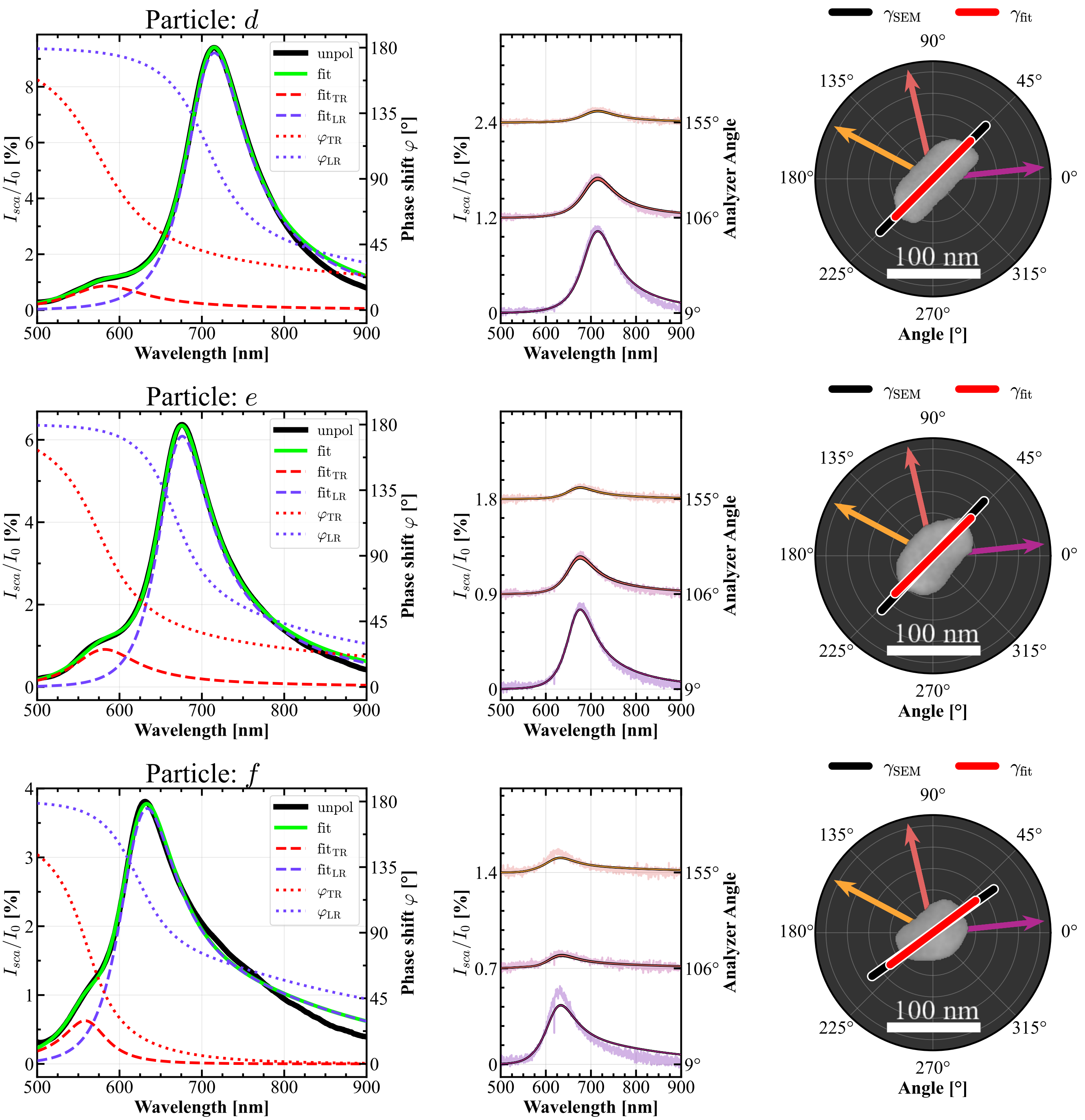

Figure S3: Particles $d - f$: (left) Unpolarized spectra with their respective double Lorentzian fit and dipole phase responses, (center) polarimetric measurements at the analyzer angles 9°, 106°, and 155°, and (right) the angles γ_{SEM} and γ_{fit} determined by SEM micrograph evaluation with the presented algorithm and cohesive fitting of the polarimetric measurements overlaid with the SEM particle.

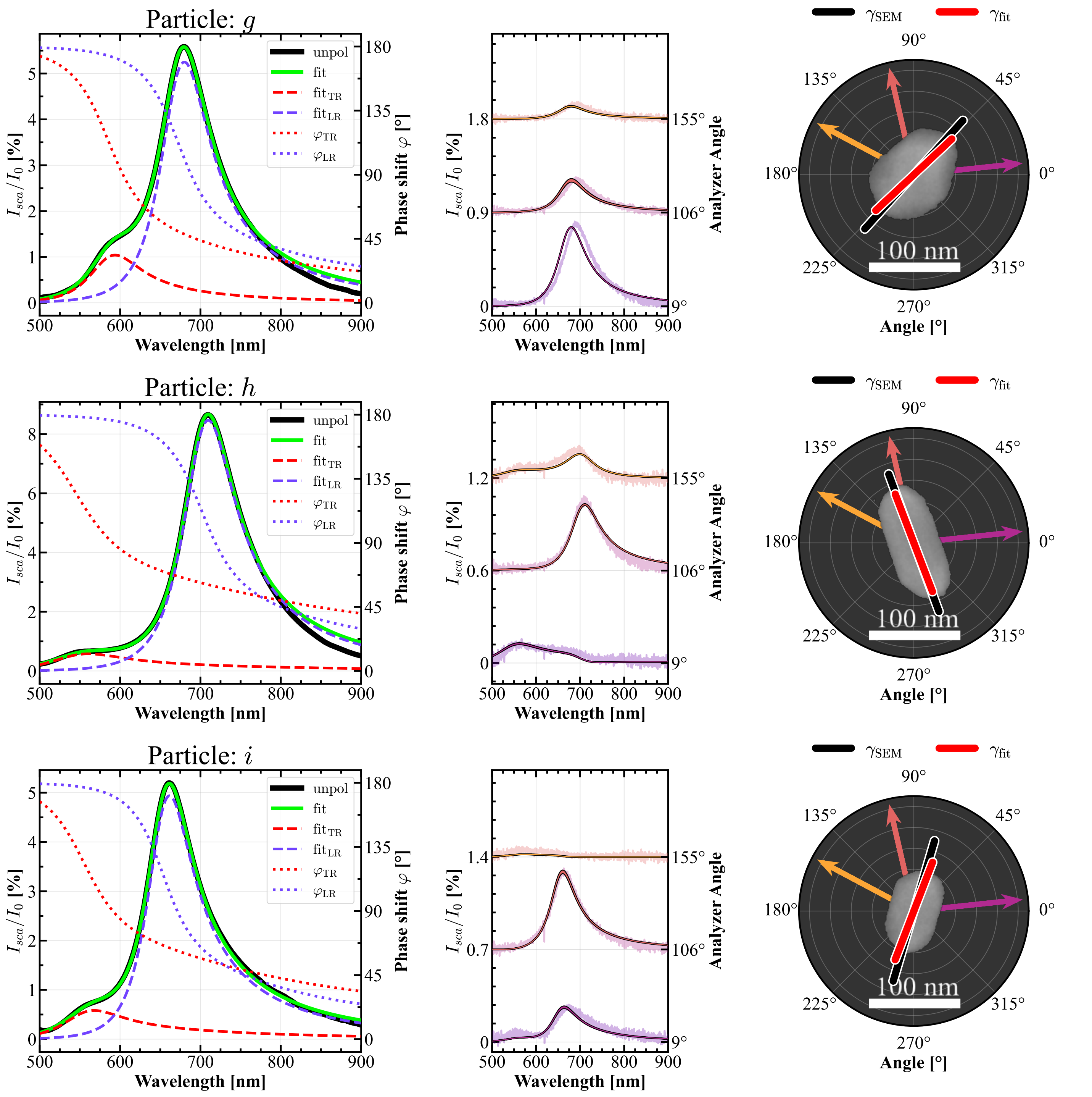

Figure S4: Particles $g - i$: (left) Unpolarized spectra with their respective double Lorentzian fit and dipole phase responses, (center) polarimetric measurements at the analyzer angles 9°, 106°, and 155°, and (right) the angles γ_{SEM} and γ_{fit} determined by SEM micrograph evaluation with the presented algorithm and cohesive fitting of the polarimetric measurements overlaid with the SEM particle.

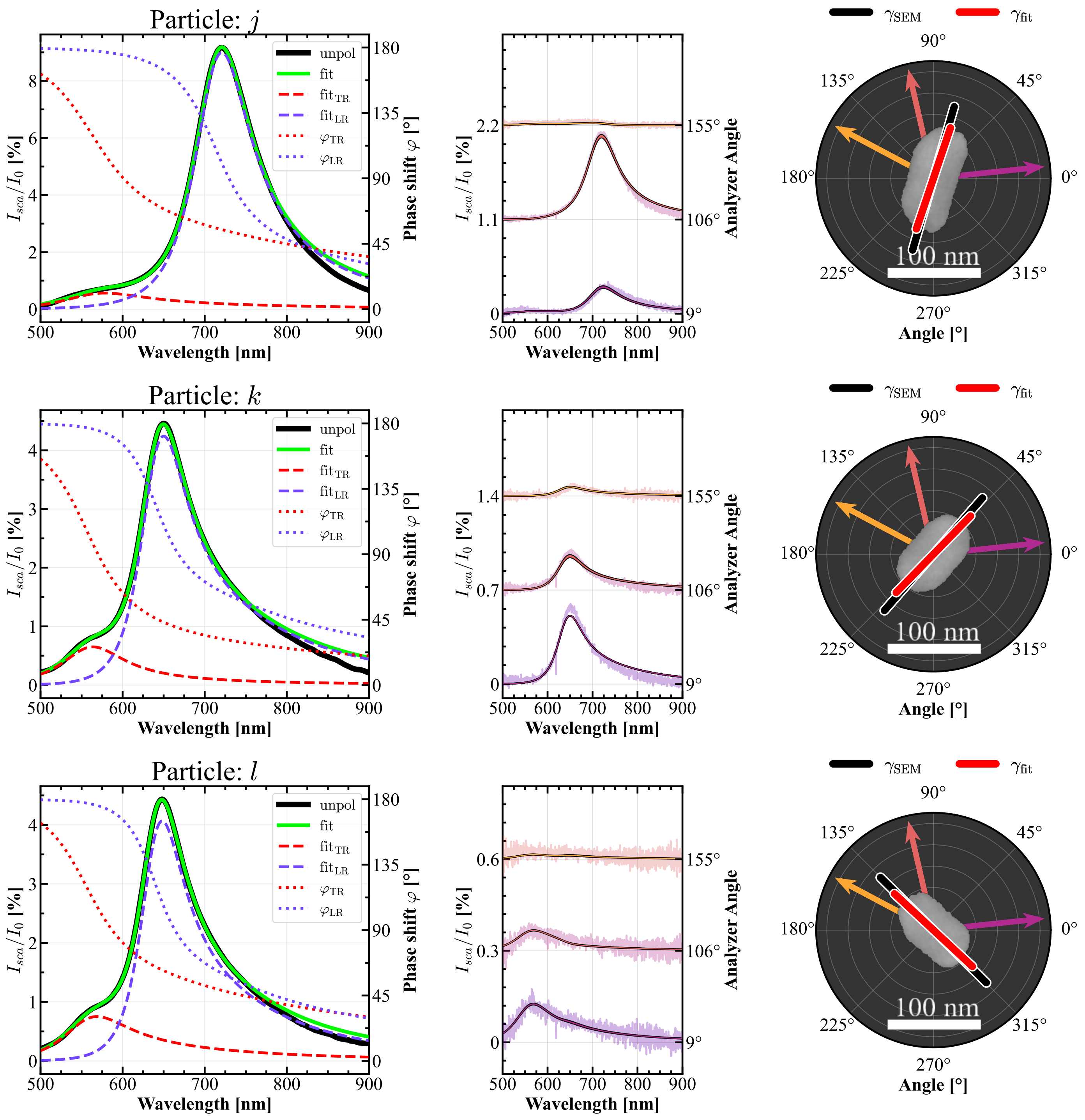

Figure S5: Particles $j - l$: (left) Unpolarized spectra with their respective double Lorentzian fit and dipole phase responses, (center) polarimetric measurements at the analyzer angles 9°, 106°, and 155°, and (right) the angles γ_{SEM} and γ_{fit} determined by SEM micrograph evaluation with the presented algorithm and cohesive fitting of the polarimetric measurements overlaid with the SEM particle.

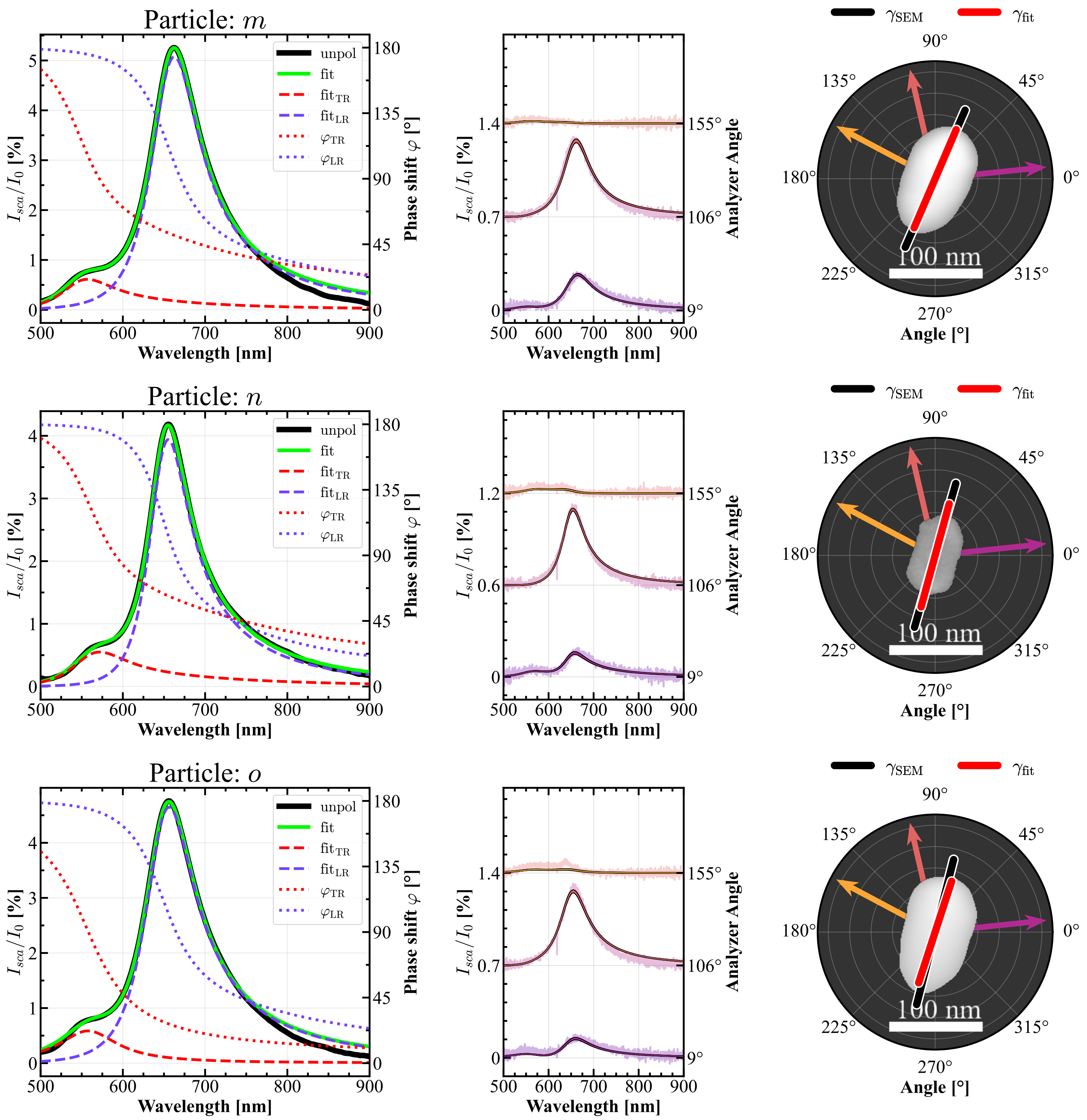

Figure S6: Particles $m-o$: (left) Unpolarized spectra with their respective double Lorentzian fit and dipole phase responses, (center) polarimetric measurements at the analyzer angles 9°, 106°, and 155°, and (right) the angles γ_{SEM} and γ_{fit} determined by SEM micrograph evaluation with the presented algorithm and cohesive fitting of the polarimetric measurements overlaid with the SEM particle.

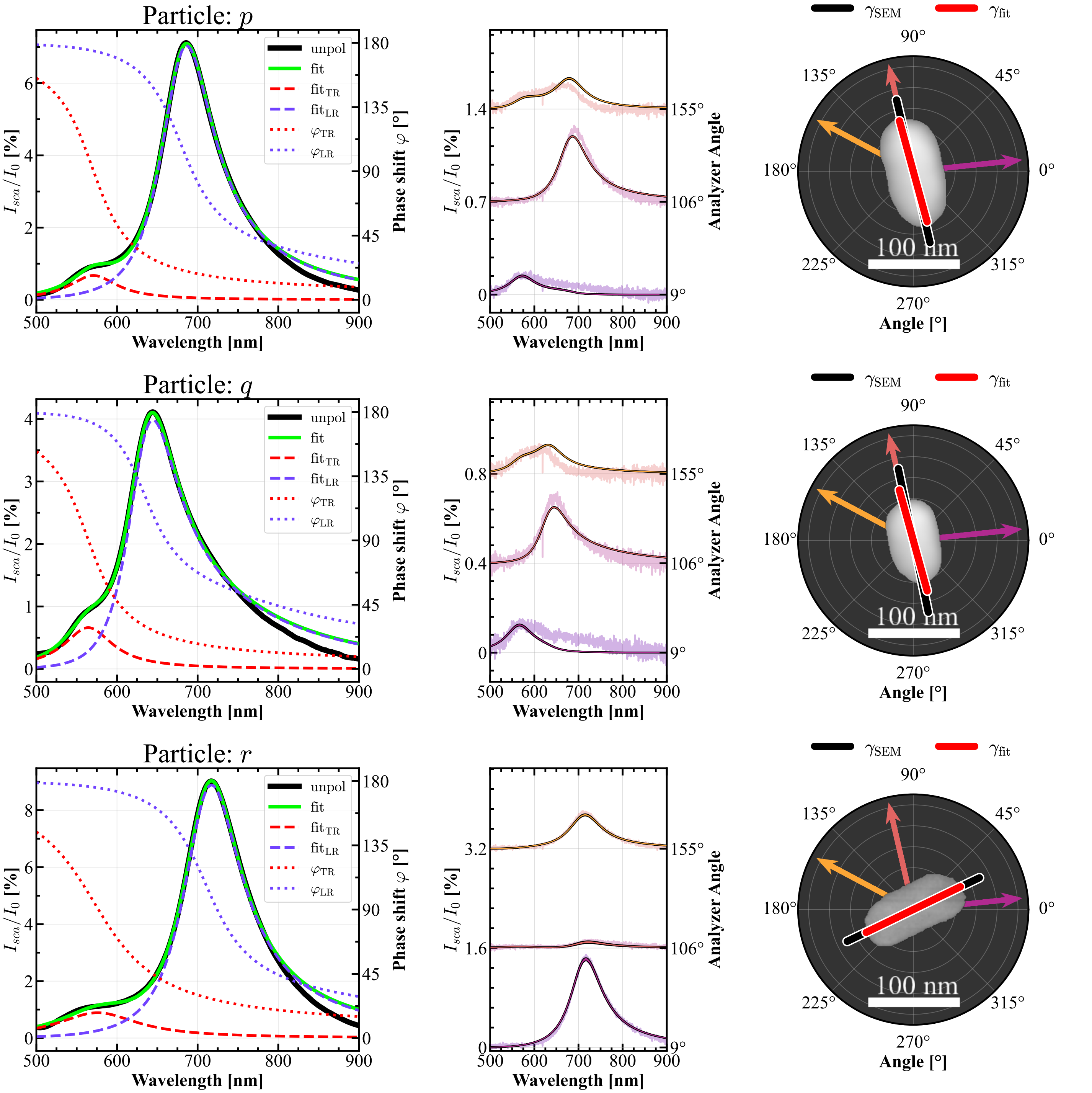

Figure S7: Particles $p - r$: (left) Unpolarized spectra with their respective double Lorentzian fit and dipole phase responses, (center) polarimetric measurements at the analyzer angles 9°, 106°, and 155°, and (right) the angles γ_{SEM} and γ_{fit} determined by SEM micrograph evaluation with the presented algorithm and cohesive fitting of the polarimetric measurements overlaid with the SEM particle.

References

[1] A. L. Stancik, E. B. Brauns, *Vib. Spectrosc.* **2008**, *47*, 1 66.

[2] A. Mildner, A. Horrer, P. Weiss, S. Dickreuter, P. C. Simo, D. Gérard, D. P. Kern, M. Fleischer, *ACS Nano* **2023**, *17*, 24 25656.

[3] G. Bradski, *Dr. Dobb's Journal of Software Tools* **2000**, *120* 122.
